# 3-Aminobenzamide Blocks MAMP-Induced Callose Deposition Independently of Its Poly(ADPribosyl)ation Inhibiting Activity

**DOI:** 10.3389/fpls.2018.01907

**Published:** 2018-12-19

**Authors:** Brian D. Keppler, Junqi Song, Jackson Nyman, Christian A. Voigt, Andrew F. Bent

**Affiliations:** ^1^Department of Plant Pathology, University of Wisconsin–Madison, Madison, WI, United States; ^2^Phytopathology and Biochemistry, Biocenter Klein Flottbek, University of Hamburg, Hamburg, Germany

**Keywords:** callose, 3-aminobenzamide, poly(ADP-ribosyl)ation, poly(ADP-ribose) polymerase, PMR4

## Abstract

Cell wall reinforcement with callose is a frequent plant response to infection. Poly(ADP-ribosyl)ation is a protein post-translational modification mediated by poly(ADP-ribose) polymerases (PARPs). Poly(ADP-ribosyl)ation has well-known roles in DNA damage repair and has more recently been shown to contribute to plant immune responses. 3-aminobenzamide (3AB) is an established PARP inhibitor and it blocks the callose deposition elicited by flg22 or elf18, two microbe-associated molecular patterns (MAMPs). However, we report that an Arabidopsis *parp1parp2parp3* triple mutant does not exhibit loss of flg22-induced callose deposition. Additionally, the more specific PARP inhibitors PJ-34 and INH_2_BP inhibit PARP activity in Arabidopsis but do not block MAMP-induced callose deposition. These data demonstrate off-target activity of 3AB and indicate that 3AB inhibits callose deposition through a mechanism other than poly(ADP-ribosyl)ation. *POWDERY MILDEW RESISTANT 4 (PMR4)* is the callose synthase responsible for the majority of MAMP- and wound-induced callose deposition in Arabidopsis. 3AB does not block wound-induced callose deposition, and 3AB does not reduce the *PMR4* mRNA abundance increase in response to flg22. Levels of PMR4-HA protein increase in response to flg22, and increase even more in flg22 + 3AB despite no callose being produced. The callose synthase inhibitor 2-deoxy-D-glucose does not cause similar impacts on PMR4-HA protein levels. Beyond MAMPs, we find that 3AB also reduces callose deposition induced by powdery mildew (*Golovinomyces cichoracearum)* and impairs the penetration resistance of a *PMR4* overexpression line. 3AB thus reveals pathogenesis-associated pathways that activate callose synthase enzymatic activity distinct from those that elevate *PMR4* mRNA and protein abundance.

## Introduction

Plants face numerous potential pathogen invaders, yet they are able to effectively prevent the large majority of these encounters from progressing to disease. Once a microbe has overcome preformed physical or chemical barriers in the plant, this disease resistance is due in large part to the plant’s innate immune system. Plants have evolved pattern recognition receptors (PRRs) that induce defense responses after recognizing certain characteristic compounds of microbes, known as microbe- or pathogen-associated molecular patterns (MAMPs, or PAMPs) (Jones and Dangl, 2006; Boller and Felix, 2009). Upon the binding of MAMPs to PRRs, an innate immune response known as pattern-triggered immunity (PTI) is initiated. PTI involves a variety of plant defense responses, including deposition of the β-(1,3)-glucan callose at plant cell walls (Dodds and Rathjen, 2010; Macho and Zipfel, 2014; Li et al., 2016).

Callose serves a variety of specialized functions in the cell walls of higher plants. Callose is one of the most abundant compounds in papillae, the cell wall thickenings formed in response to pathogen attack (Aist, 1976; Ellinger and Voigt, 2014; Schneider et al., 2016). In addition to its roles in pathogen defense, callose plays critical roles in pollen development (Dong et al., 2005; Enns et al., 2005; Shi et al., 2015), formation of the cell plate during cytokinesis (Chen et al., 2009; Thiele et al., 2009; Park et al., 2014), and regulation of cell-to-cell trafficking through the plasmodesmata (Lee and Lu, 2011; Vaten et al., 2011; Cui and Lee, 2016). The Arabidopsis genome encodes a total of twelve callose synthase enzymes (Richmond and Somerville, 2000; Hong et al., 2001). PMR4 (POWDERY MILDEW RESISTANT 4; also known as GLUCAN SYNTHASE-LIKE 5 [GSL5] and CALS12) accounts for nearly all wound- and pathogen-induced callose, as both responses are absent in *pmr4* mutants (Jacobs et al., 2003; Nishimura et al., 2003; Clay et al., 2009; Luna et al., 2011).

PMR4 was initially identified in a screen to identify mutants with increased resistance to an Arabidopsis powdery mildew pathogen, *Erysiphe cichoracearum* (now known as *Golovinomyces cichoracearum*) (Vogel and Somerville, 2000). Given that callose deposition was regarded as a cell wall reinforcement to prevent further pathogen ingress, the finding that a mutant lacking callose was more resistant to a pathogen was counterintuitive and initially cast doubt on the importance of callose deposition in the plant innate immune response, but *pmr4* mutants exhibit constitutively elevated or primed defenses that apparently account for the increased resistance (Nishimura et al., 2003). More recently, overexpression of *PMR4* was shown to enhance callose deposition and also increases resistance to adapted powdery mildew (Ellinger et al., 2013). Despite the ubiquitous presence of callose deposition in plant innate immune responses, much remains to be understood about the regulation of pathways between MAMP perception by PRRs or wounding and the PMR4 callose synthase, including the activation of PMR4 and its relocalization to sites of pathogen attack (Ellinger and Voigt, 2014; Schneider et al., 2016).

Poly(ADP-ribosyl)ation is a post-translational modification of proteins in which chains of ADP-ribose are added to a target protein by enzymes known as poly-ADP-ribose polymerases (PARPs) (Gibson and Kraus, 2012). Poly-ADP ribose chains can vary in length and branching. These poly-ADP-ribose moieties can be removed by poly-ADP-ribose glycohydralase (PARG) enzymes. Poly(ADP-ribosyl)ation has been studied extensively in animal systems because of its involvement in DNA damage repair and an increasing number of other cellular stress responses (Schreiber et al., 2006; Kalisch et al., 2012) and due to the potential use of therapeutic PARP inhibitors in cancer treatment (Virag and Szabo, 2002; Ellisen, 2011). Poly(ADP-ribosyl)ation and related processes, including DNA damage, also have been demonstrated to play important roles in plant immune system responses to infection (Lucht et al., 2002; Briggs and Bent, 2011; Yan et al., 2013; Yao et al., 2013; Song and Bent, 2014; Feng et al., 2015; Song et al., 2015; Feng et al., 2016a,b; Briggs et al., 2017).

The Arabidopsis genome encodes three PARP genes similar in structure to those found in mammalian systems, namely PARP1, PARP2, and PARP3 (Briggs and Bent, 2011; Lamb et al., 2012). PARP2 plays a more important role than PARP1 in DNA damage responses and plant immune responses (Song et al., 2015), while PARP3 is expressed primarily in seeds and may promote seed viability (Rissel et al., 2014). In addition, six plant-specific proteins with PARP domains were previously identified: the SRO (SIMILAR TO RCD ONE) family, consisting of the founding member RCD1 as well the related SRO1 through SRO5 (Overmyer et al., 2000; Ahlfors et al., 2004). RCD1 has been confirmed experimentally to lack PARP activity and bioinformatics-based sequence analysis indicated the remaining members are unlikely to possess PARP activity (Jaspers et al., 2010). However, wheat SRO1 was confirmed to exhibit PARP activity and play a role in abiotic stress resistance (Liu et al., 2014).

In order to study poly(ADP-ribosyl)ation and/or overcome the potential functional redundancy of multiple PARP enzymes, chemical inhibitors of PARP are often utilized. 3-aminobenzamide (3AB) is a well characterized inhibitor of poly(ADP-ribosyl)ation that has been used in studies with mammalian systems since the early 1980s (Purnell and Whish, 1980; Virag and Szabo, 2002; Ellisen, 2011). Notably, 3AB blocks some plant innate immune responses while others remain intact. In general, the early steps following recognition of a MAMP remain intact, while later events are disrupted (Adams-Phillips et al., 2010). Specifically, expression of early MAMP-induced genes such as *WRKY29* and *FRK1* are still upregulated and reactive oxygen species (ROS) production still occurs even in the presence of 3AB. However, flg22-induced callose deposition, which is typically apparent 12 to 24 h following MAMP perception, is inhibited by 3AB (Adams-Phillips et al., 2010).

Here, we further characterize the inhibition of MAMP-induced callose deposition by 3AB and identify impacts on powdery mildew-induced callose deposition. Surprisingly, we find that the inhibition of callose deposition by 3AB is largely independent of poly(ADP-ribosyl)ation. However, 3AB does impact PMR4 protein abundance in the presence of flg22, suggesting novel mechanisms of PMR4 regulation. Hence 3AB is a valuable chemical genetic tool to probe the unique pathways that regulate pathogenesis-related plant callose deposition.

## Materials and Methods

### Plant Material, Growth Conditions, Inhibitors, Wounding, and Inoculations

Arabidopsis (*Arabidopsis thaliana*) seedlings were grown for flg22 callose assays as previously described, with some modifications (Clay et al., 2009; Luna et al., 2011). Briefly, 15 seeds per well were distributed into each well of a 12-well plate. 1 ml of liquid Murashige-Skoog (MS) media was added to each well, plates were sealed with micropore tape and cold treated for 2–3 days at 4°C. Following cold treatment, plates were moved to growth chambers at 22°C under 16-h light/8-h dark cycles. The media was replaced on day 7 and then on day 9 seedlings were treated with elicitors (1 μM flg22) and/or inhibitors [the PARP inhibitors 1 mM 3AB, 1 mM 3MB, 100 μM PJ-34, 100 μM INH_2_BP, or the callose synthase inhibitor 3 mM 2-deoxy-D-glucose (2-DDG)]. 3MB is 3-methoxybenzamide. PJ-34 is 2-(dimethylamino)-*N*-(6-oxo-5,6-dihydrophenanthridin-2-yl)acetamide hydrochloride; CAS number 344458-15-7. INH_2_BP is 5-iodo-6-amino-1,2-benzopyrone; CAS number 137881-27-7. Dose–response experiments with inhibitors were performed prior to their use with flg22 and established active but non-lethal dosing for PJ-34 and INH_2_BP by growing Arabidopsis seedlings on plates with 10 nM, 100 nM, 1 μM, 10 μM, 100 μM, or 1 mM of the inhibitor. Increased seedling death was first evident with concentrations of 1 mM PJ-34 or 1 mM INH_2_BP. For wounding, each cotyledon was gently compressed once by full release of reverse-action tweezers, to provide consistent wounding force across samples. Unless otherwise noted, seedlings were collected after 24 h of treatment and examined for callose as described below.

For powdery mildew experiments, Arabidopsis wild-type (Columbia Col-0), *pmr4* (allele 1; Nishimura et al., 2003) and the *PMR4* overexpression lines *35S:PMR4-GFP* (Ellinger et al., 2013) as well as the powdery mildew *Golovinomyces cichoracearum* (*Gc*, strain UCSC1) were cultivated as described in Stein et al. (2006). Three-week-old plants were used in all experiments. 3AB was applied to adult leaves by infiltration into the leaf mesophyll using a syringe with no needle; callose and PMR4-GFP data were then taken from leaf sites not on or directly adjacent to the site of syringe contact with the leaf.

The previously described *parp1-2* (GABI_382F01) *parp2-1* (GABI_420G03) double mutant (Song et al., 2015) was crossed with a *parp3* (SALK_108092) mutant and progeny were genotyped to identify a *parp1parp2parp3* triple mutant. GFP-PEN1 and PEN3-GFP lines were previously described and kindly provided by William Underwood (Collins et al., 2003; Stein et al., 2006).

### Flg22-Responsive Callose Deposition and Epifluorescence Microscopy

Following elicitor/inhibitor treatment, seedlings were fixed in an FAA solution (10% formaldehyde, 5% acetic acid, and 50% ethanol) overnight, cleared in 95% ethanol, and stained with aniline blue (0.01% aniline blue in 67 mM K_2_HPO_4_ with pH adjusted to 12). The stained seedlings were visualized with an Olympus BX60 Epifluorescence Microscope and images of entire cotyledons were captured with an Olympus DP73 camera. At least 12 cotyledons were imaged per line per treatment and callose deposits were quantified automatically using ImageJ software and compared to total cotyledon area in order to calculate the percent area with callose deposits. Aniline blue staining for cytological analyses of powdery mildew treated samples followed the protocol of Stein et al. (2006).

### Powdery Mildew-Responsive Callose Deposition and Confocal Microscopy

Confocal microscopy of callose deposition at infection sites and localization of PMR4-GFP after infection followed the description in Ellinger et al. (2014) using the confocal laser-scanning microscope LSM 780 (Carl Zeiss MicroImaging GmbH) and the 10× objective (EC Plan-Neofluar 10x/0.30 M27) for overview images and the 63x water-immersion objective (C-Apochromat 63x/1.20 W Korr M27) for Z series of infection sites. Aniline blue was excited at 405 nm and emission detected through a 410–485 nm bandpass filter; GFP was excited at 488 nm and emission detected through a 499–560 nm bandpass filter. 3D surface rendering of Z series were generated with the ZEN 2010 operating software (Carl Zeiss MicroImaging GmbH).

### Generating Tagged PMR4 Lines

The full-length genomic sequence of *PMR4* along with the 2000 base pair upstream promoter region and excluding the stop codon were amplified (5′-CGGGCAAGTTCCAAAGTTTTG-3′ and 5′-GACATCGCCTTTTGATTTCTTCC-3′) by PCR and cloned into the pCR8/GW/TOPO vector according to the manufacturer’s protocol (Thermo Fisher Scientific). The fragment was then recombined by LR cloning into pGWB13 (for C-terminal HA tag) and pGWB4 (for C-terminal GFP tag) (Nakagawa et al., 2007), which were then transformed into Arabidopsis *pmr4-1* mutant lines by *Agrobacterium*-mediated floral dip transformation (Clough and Bent, 1998). Transformants were selected by antibiotic selection and all experiments were performed on homozygous T3 plants.

### RNA Extraction and Gene Expression Analysis

RNA was extracted using TRIzol reagent (Thermo Fisher Scientific) with DNA removed with the DNA-free DNA Removal Kit (Thermo Fisher Scientific). RNA concentrations were determined using the NanoDrop 1000 spectrophotometer (Thermo Fisher Scientific). cDNA was synthesized from RNA using iScript cDNA synthesis kit (Bio-Rad) according to the manufacturer’s protocol. Quantitative PCR was performed with a CFX96 real-time PCR detection system (Bio-Rad). Primers used for PMR4 (5′-CTGGAATGCTGTTGTCTCTGTTG-3′ and 5′-TCGCCTTTTGATTTCTTCCCAGT-3′) were previously described (Jacobs et al., 2003). Primers for TIP41-like family protein (At4g34270) were used as an internal control (Czechowski et al., 2005). The delta-delta Ct method (2^-ΔΔCt^ method) was used to calculate the relative gene expression of samples.

### Protein Extraction and Protein Immunoblot Analysis

Total protein extracts were prepared from Arabidopsis plants in extraction buffer (50 mM Tris-HCl pH 7.5, 150 mM NaCl, 5 mM EDTA, 0.5% Triton X-100, 10% glycerol, and Sigma-Aldrich plant protease inhibitor cocktail at 1:100) as described previously (Song et al., 2015). Because of the multiple transmembrane domains of PMR4, samples were heated in sample buffer at 37°C for 30 min to avoid protein aggregation. Following protein separation by SDS-PAGE, Western blot analysis was performed with anti-poly(ADP-ribose) (anti-PAR), anti-HA, or anti-GFP antibodies.

### Statistical Analyses

Unless otherwise noted, descriptive statistics including the mean and SD along with the Bonferroni–Holm range test for multiple comparison procedure in conjunction with an ANOVA were used to determine significant differences. *P* < 0.05 was considered significant.

## Results

### 3AB Blocks flg22-Induced Callose but Not Wound-Induced Callose

**FIGURE 1:**
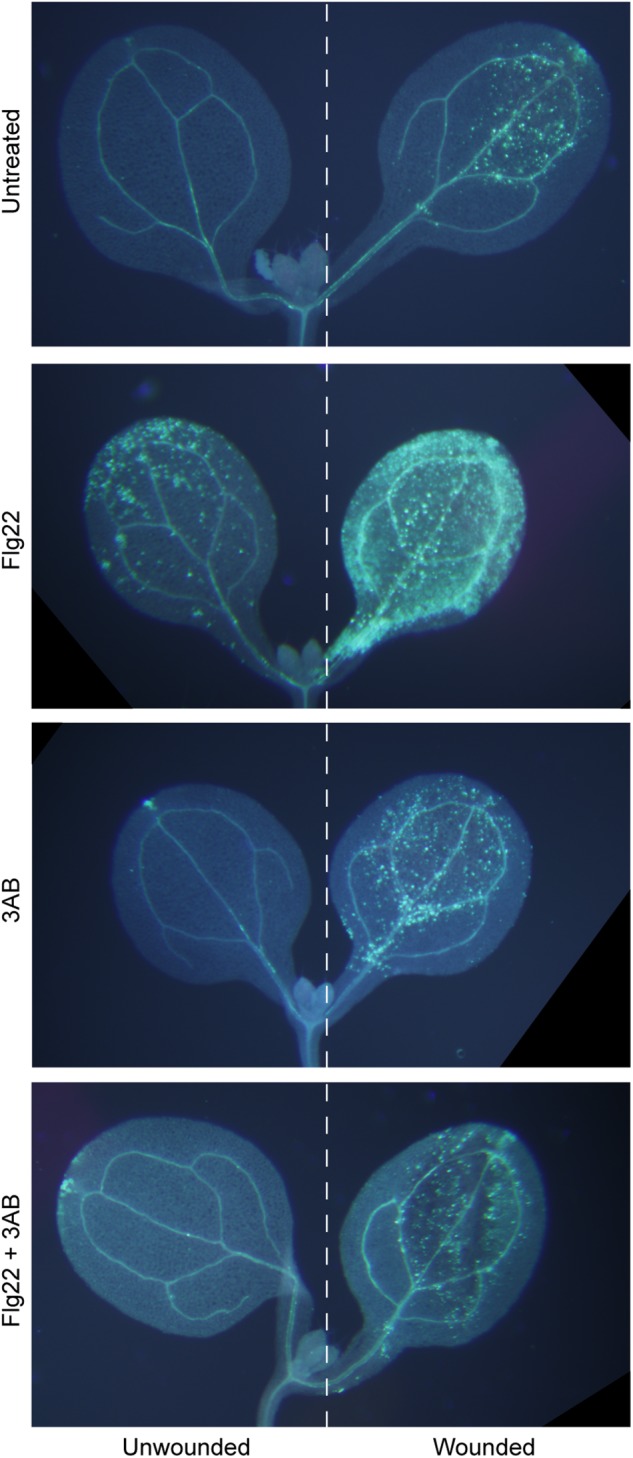
The poly(ADP-ribosyl)ation inhibitor 3AB blocks callose deposition in response to flg22, but not in response to wounding. Intact Arabidopsis seedlings were treated with flg22 and/or 3AB as described, and also were wounded on one cotyledon (right half of figure) but not the other (left half). Images are from within one experiment, and are representative of at least 38 seedlings per treatment from three independent experiments.

In Arabidopsis, flg22 and wound-induced callose are the product of the same PMR4 callose synthase enzyme, as both are absent in a *pmr4* mutant (Jacobs et al., 2003; Nishimura et al., 2003; Luna et al., 2011). Interestingly, while 3AB blocks flg22-induced callose, wound-induced callose is not detectably affected by 3AB (Adams-Phillips et al., 2010). We first performed an extended version of that experiment, combining flg22 and wounding treatments on some cotyledons, and, imaging seedlings in which one cotyledon was wounded and the adjacent cotyledon on the same seedling was left unwounded. Even within a single seedling exposed to flg22 and with one cotyledon wounded, we found that 3AB blocks the flg22-induced callose but not the wound-induced callose (Figure 1). A separable and additive or synergistic phenomenon was also reproducibly observed, wherein leaves treated with both flg22 and wounding consistently produced a much stronger callose response than either treatment separately (Figure 1). Addition of 3AB reduced the callose levels in leaves exposed to both flg22 and wounding back down to the levels typically observed for wounding in the absence of flg22 (Figure 1). This intriguing specificity strongly suggests that 3AB does not inhibit callose synthase activity directly. Rather, the target of 3AB is likely to be upstream of PMR4 callose synthase catalytic activity and specific to flg22-activated or other pathogenesis-associated callose deposition pathways, but downstream of initial MAMP detection, because early plant innate immune responses such as MAP kinase activation and ROS production were previously shown to occur in even the presence of 3AB (Adams-Phillips et al., 2010).

### *parp1parp2parp3* Triple Mutants Still Produce flg22-Induced Callose

**FIGURE 2:**
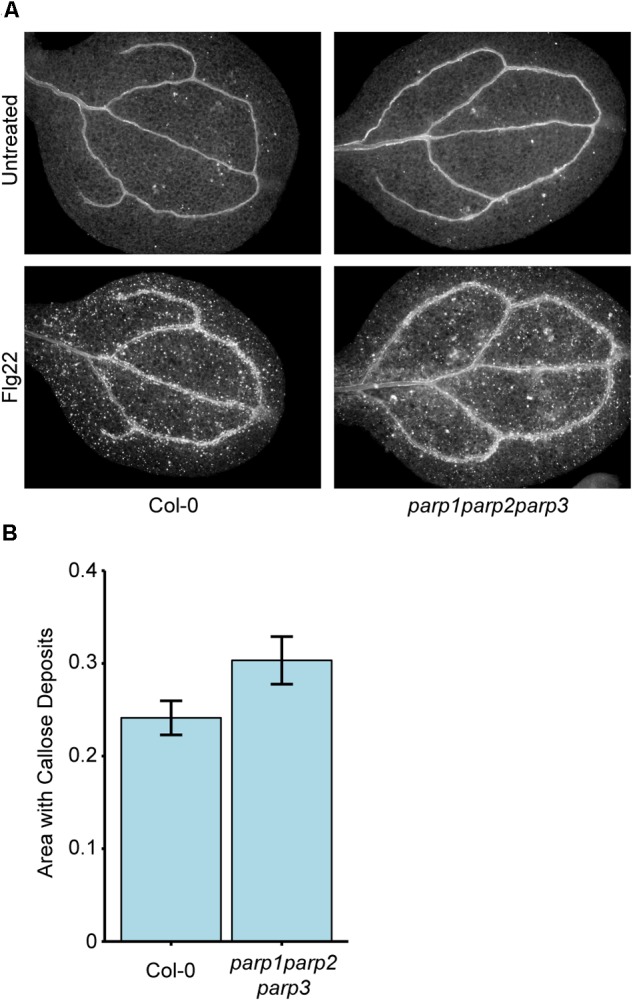
Col-0 and *parp1parp2parp3* triple mutants produce similar levels of flg22-induced callose. **(A)** Representative images from Col-0 and the *parp1parp2parp3* triple mutant untreated or treated with flg22. **(B)** Quantification of callose in cotyledons treated with flg22 as in **(A)**. Area represents total area with callose divided by total cotyledon area. Experiment repeated three times with twelve cotyledons per line per experiment. Error bars indicate standard error of the mean. No significant differences were observed (*t*-test, *p* > 0.05).

Because 3AB is a well-characterized inhibitor of PARP-mediated poly(ADP-ribosyl)ation (Purnell and Whish, 1980; Virag and Szabo, 2002; Ellisen, 2011), we hypothesized that 3AB blocks callose deposition by inhibiting the activity of one or more of the three enzymes with predicted PARP activity in Arabidopsis, namely PARP1, PARP2, and PARP3, and that this PARP activity is required for flg22-induced callose deposition. We constructed a *parp1parp2parp3* triple mutant to investigate the role of all three PARP enzymes in callose deposition. Surprisingly, the *parp1parp2parp3* triple mutant does not exhibit a loss of flg22-induced callose as is observed with 3AB, or even a reduction, and if anything exhibits a slight (but statistically insignificant) increase in callose deposition compared to wild type (Figure 2). We have demonstrated that the *parp1parp2* double mutant that was used to make this triple mutant already exhibits little or no poly(ADP-ribosyl)ation activity (Song et al., 2015). The differing callose response phenotypes with the PARP inhibitor 3AB as opposed to the *parp1parp2parp3* triple mutant brings up known issues about the specificity of 3AB as an inhibitor of poly(ADP-ribosyl)ation (Virag and Szabo, 2002).

### INH_2_BP and PJ-34 Are Bona Fide PARP Inhibitors in Arabidopsis but Do Not Impact Callose Deposition

**FIGURE 3:**
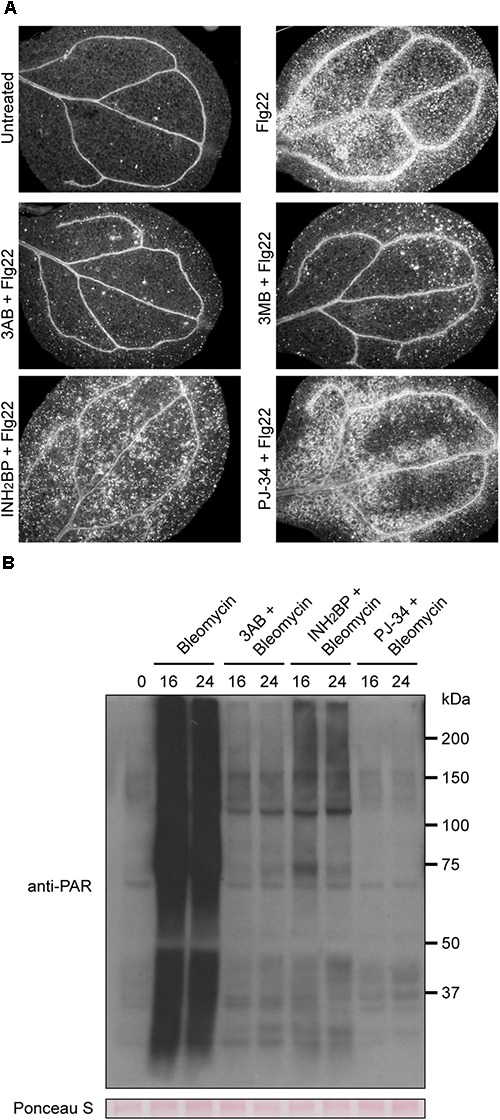
INH_2_BP and PJ-34 inhibit PARP activity but do not impact flg22-induced callose deposition. **(A)** Representative images of cotyledons treated with flg22 alone, or flg22 with the inhibitors 3AB, 3MB, INH_2_BP, or PJ-34. **(B)** Total protein extracts from seedlings treated with the DNA damage reagent bleomycin for 16 or 24 h with or without PARP inhibitors were analyzed by SDS-PAGE immunoblots with an anti-PAR antibody to detect poly(ADP-ribosyl)ated proteins. Equivalent loading of lanes was verified using Ponceau S stain. Experiments repeated three times with 12 cotyledons per treatment per experiment.

Other known PARP inhibitors were tested to determine if they also block flg22-induced callose deposition as is observed with 3AB. Numerous PARP inhibitors have been developed to target mammalian PARPs in recent years as research tools as well as for potential chemotherapeutic uses (Rouleau et al., 2010), but these have not previously been reported as inhibitors of plant PARPs. The more specific PARP inhibitors PJ-34, which is considered 10,000 times more potent than 3AB, and INH_2_BP, were obtained (Abdelkarim et al., 2001). PJ-34 and INH_2_BP each failed to inhibit flg22-induced callose deposition (Figure 3A). The PARP inhibitor 3MB, which is structurally related to 3AB, did reproducibly reduce flg22-induced callose deposition (Figure 3A).

In order to confirm that INH_2_BP and PJ-34 are bona fide PARP inhibitors in Arabidopsis, plants were treated with the DNA damage reagent bleomycin in the presence or absence of the inhibitors. Use of an anti-PAR antibody after bleomycin treatment showed that bleomycin strongly induces poly(ADP-ribosyl)ation of target proteins, including the PARP enzymes themselves. At either 16 or 24 h following bleomycin treatment, poly(ADP-ribosyl)ation is strongly induced. Poly(ADP-ribosyl)ation was inhibited not only by 3AB but also by INH_2_BP, and PJ-34 rendered particularly complete inhibition (Figure 3B). These results demonstrate that INH_2_BP and PJ-34 are bona fide PARP inhibitors in Arabidopsis, yet do not inhibit flg22-induced callose deposition, supporting the conclusion that the impact of 3AB on callose is independent of PARP activity.

### 3AB Does Not Reduce *PMR4* Gene Expression

**FIGURE 4:**
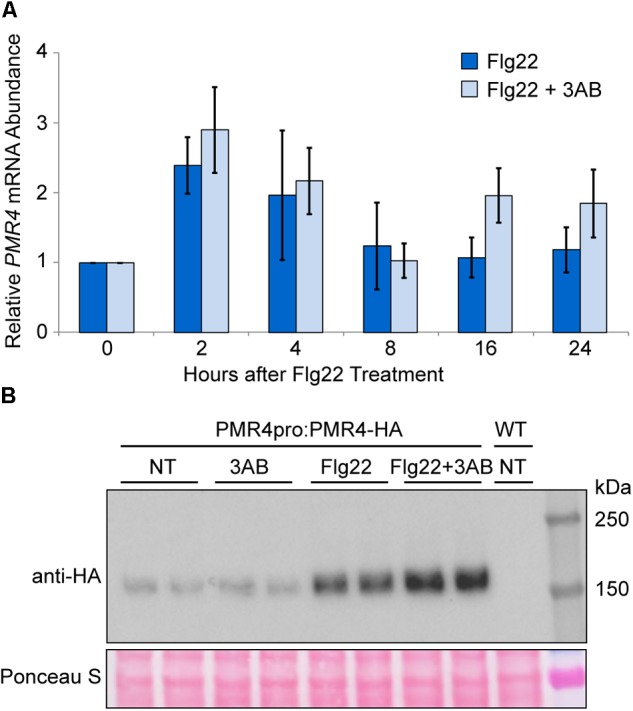
*PMR4* mRNA abundance after flg22 treatment is not reduced by 3AB, but 3AB increases the abundance of PMR4-HA protein. **(A)** qRT-PCR analysis of *PMR4* mRNA abundance at various time points following flg22 or flg22 + 3AB treatment. Data are the mean for six biologically independent samples, three each from two separate experiment dates. Error bars indicate standard error of the mean. There were no significant differences between treatments within a time point (*t*-test, *p* > 0.05). **(B)** Total protein extracts from seedlings analyzed by SDS-PAGE immunoblotting with an anti-HA antibody. Two independent plant samples are shown for each treatment. Equivalent loading of lanes was verified using Ponceau S stain. NT, no treatment; WT, Col-0 wild-type negative control. Similar results were obtained in over six independent experiments and in experiments with *PMR4pro:PMR4-GFP*.

Despite questions about the target of 3AB, its intriguing impact on callose deposition may make it a useful tool to elucidate regulatory mechanisms that control pathogenesis-responsive callose deposition. As such, we investigated the impacts of 3AB on PMR4. Quantitative real time (qRT)-PCR was utilized to monitor *PMR4* gene expression following flg22 treatment in the presence or absence of 3AB. We observed a twofold to threefold induction of *PMR4* gene expression at 2 and 4 h following flg22 treatment (Figure 4A). A similar induction of *PMR4* was observed even in the presence of 3AB, demonstrating that 3AB does not block, either directly or indirectly, *PMR4* gene expression.

### 3AB Increases PMR4-HA Protein Abundance in the Presence flg22

In order to discover impacts of 3AB on PMR4 protein levels following flg22 and/or 3AB treatments, an HA-tagged *PMR4* construct under control of the *PMR4* native promoter (*PMR4pro:PMR4-HA*) was generated and transformed into the *pmr4* mutant background. The *pmr4* mutant line transformed with *PMR4pro:PMR4-HA* regained the capacity to deposit callose in response to flg22 at wild type levels (Supplementary Figure S1). Flg22-induced callose deposition is also blocked by 3AB in this line, confirming that the construct successfully rescues the *pmr4* mutant phenotypes. Similar results were obtained with an independent GFP-tagged *PMR4pro:PMR4-GFP* transformed into the *pmr4* mutant background (Supplementary Figure S1).

The abundance of the PMR4-HA protein was monitored at 24 h after treatments with flg22 and 3AB. Consistent with observations in the above gene expression study, an increase in PMR4-HA protein levels was observed in flg22-treated samples as compared to untreated controls (Figure 4B). Intriguingly, in multiple independent experiments, samples treated with flg22 + 3AB consistently exhibited greater PMR4-HA abundance compared to those treated with flg22 alone, despite the fact that no callose is produced. One interpretation of this is that 3AB inhibits callose production during the response to flg22 and the plant responds by further upregulating *PMR4* mRNA and PMR4 protein abundance, but because 3AB through an unknown mechanism continues to inhibit callose production, no callose is produced. Because wound-induced callose is a product of the same PMR4 callose synthase but is not blocked, PMR4-HA was also monitored with 3AB and wounding. As predicted, unlike for flg22, an increase in PMR4-HA protein was not observed with 3AB and wounding compared to wounding alone (Supplementary Figure S2). We also observed that samples treated with 3AB in the absence of flg22 sometimes exhibited decreased PMR4-HA abundance as compared to untreated controls, but this phenomenon was not consistently observed across all biological replicates. Taken together, the above results indicate that one or more components of the flg22-induced signaling mechanisms that activate callose synthase enzymatic activity can be inhibited by 3AB, and are distinct from those that elevate PMR4 mRNA and protein levels.

Additional experiments assessed if the impact of 3AB on protein abundance is specific to PMR4 or if it impacts the abundance other proteins that contribute to the formation of callose-rich papillae at sites of infection. Levels of GFP-tagged PENETRATION1 and PENETRATION3 (GFP-PEN1 and PEN3-GFP) were therefore examined. *PEN1* encodes a syntaxin, while *PEN3* encodes an ABC transporter, and each is required for an independent pathway leading to penetration resistance to powdery mildew (Collins et al., 2003; Stein et al., 2006). We observed that PEN3-GFP is strongly induced by flg22, but additional PEN3-GFP protein is not induced by 3AB as is observed with PMR4-HA (Supplementary Figure S3). Similarly, GFP-PEN1 levels remained relatively constant whether treated with flg22 or 3AB (Supplementary Figure S3).

### Inhibition of PARP Activity or Callose Synthase Activity Do Not Alter PMR4 Levels

**FIGURE 5:**
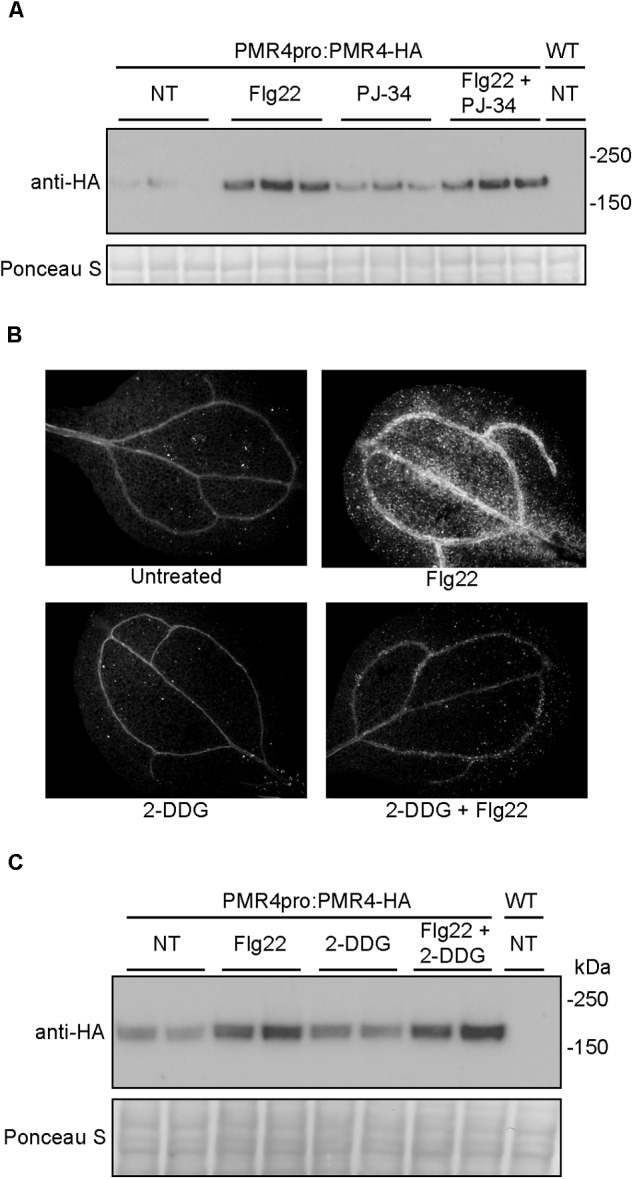
Neither the more potent and specific PARP inhibitor PJ-34 nor the callose synthase inhibitor 2-DDG cause alterations in PMR4-HA abundance. **(A)** Total protein extracts from seedlings treated with PJ-34 and/or flg22 were analyzed by SDS-PAGE immunoblotting with an anti-HA antibody. Three biologically independent plant samples are shown for each treatment. Similar results were obtained in two separate experiments (total *n* = 6 per treatment). **(B)** Representative cotyledons untreated or treated with flg22, 3 mM 2-DDG, or 3 mM 2-DDG and flg22. **(C)** Total protein extracts from seedlings treated with 2-DDG and/or flg22 were analyzed by SDS-PAGE immunoblotting with an anti-HA antibody. Two biologically independent plant samples are shown for each treatment. Equivalent loading of lanes was verified using Ponceau S stain. NT, no treatment; WT, Col-0 wild-type negative control. Similar results were obtained in two separate experiments (total *n* = 4 per treatment).

Results described above indicate that the loss of callose deposition caused by 3AB is unlikely to be due to PARP inhibition, but we also wanted to exclude the possibility that the changes in PMR4 abundance were due to inhibition of poly(ADP-ribosyl)ation. In order to do so, the impact of the PARP inhibited PJ-34 was also examined. The increase in PMR4-HA observed with flg22 + 3AB is not apparent when seedlings are treated with flg22 + PJ-34 (Figure 5A). Given that PJ-34 is a more potent and specific PARP inhibitor than 3AB (Abdelkarim et al., 2001), and an inhibitor that more effectively blocks DNA damage induced poly(ADP-ribosyl)ation in Arabidopsis (Figure 3B), this provides evidence that the impact of 3AB on both callose deposition and PMR4 protein abundance are independent of poly(ADP-ribosyl)ation.

2-Deoxy-D-glucose is a non-metabolizable form of glucose widely used as a direct inhibitor of callose synthase activity (Jaffe and Leopold, 1984; Bayles et al., 1990; Li et al., 2012). At a concentration of 3 mM 2-DDG, flg22-induced callose deposition was blocked (Figure 5B). However unlike 3AB, 2-DDG treatment did not alter PMR4-HA protein levels (Figure 5C), suggesting that the mechanism of elevation of PMR4 protein abundance in flg22 + 3AB treatments is not solely due to the disruption of callose synthase activity after flg22 treatment.

### 3AB Inhibits Some Powdery Mildew-Induced Callose Deposition

**FIGURE 6:**
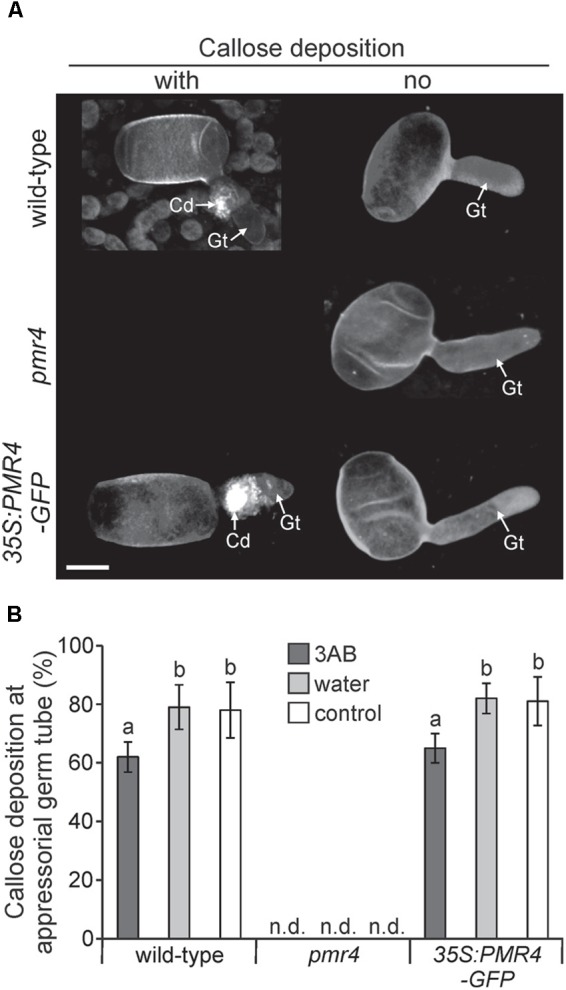
3AB reduces the frequency of callose deposition at powdery mildew infection sites. Three-week-old overexpression line *35S:PMR4-GFP, pmr4* and wild-type plants were inoculated with a compatible powdery mildew (*G. cichoracearum*) isolate 2 h after infiltration with 10 mM 3AB. Leaves infiltrated with water served as mock control, leaves without infiltration as infection control. **(A)** Representative micrographs of sites with and without (no) pathogen-induced callose deposition (fluorescence by aniline blue staining) at appressorial germ tubes (Gt) at an early time-point 6 h post-inoculation (hpi). Micrographs are 3D projections taken by confocal laser-scanning microscopy. *pmr4* samples all exhibited no callose deposition. Scale bar = 10 μM. **(B)** Percent of samples showing callose deposition at appressorial germ tubes at 6 hpi. Bars with shared letter not significantly different (*P* < 0.05 by Bonferroni–Holm’s test). Error bars represent ± SD, and *n* = 50 of four independent leaves. n.d., callose deposition not detected.

While 3AB has been shown to block flg22- and elf18-induced callose deposition (Adams-Phillips et al., 2008), we sought to understand if 3AB also blocks the callose deposited in response to Arabidopsis powdery mildew. Following leaf infiltration of 10 mM 3AB or water only, plants were inoculated and callose deposition was examined 6 h post inoculation (hpi) in Col-0 wild-type, *pmr4* mutants, as well as *35S:PMR4-GFP* overexpression lines (Ellinger et al., 2013). In both Col-0 and the overexpression lines, the presence of callose at powdery mildew appressorial germ tubes was quantitatively but significantly reduced by 3AB (Figure 6). In controls that received no 3AB callose was observed at close to 80% of powdery mildew sites, but was reduced to only 60% by 3AB treatment. Intriguingly, 3AB had an all-or-nothing effect on callose deposition after powdery mildew infection, with fewer sites showing callose deposition but no indication that the quantity of callose was altered at sites that had callose deposition. Controls confirmed the previous finding that in the PMR4 overexpression line a callose response occurs due to any infiltration (Supplementary Figure S4).

**FIGURE 7:**
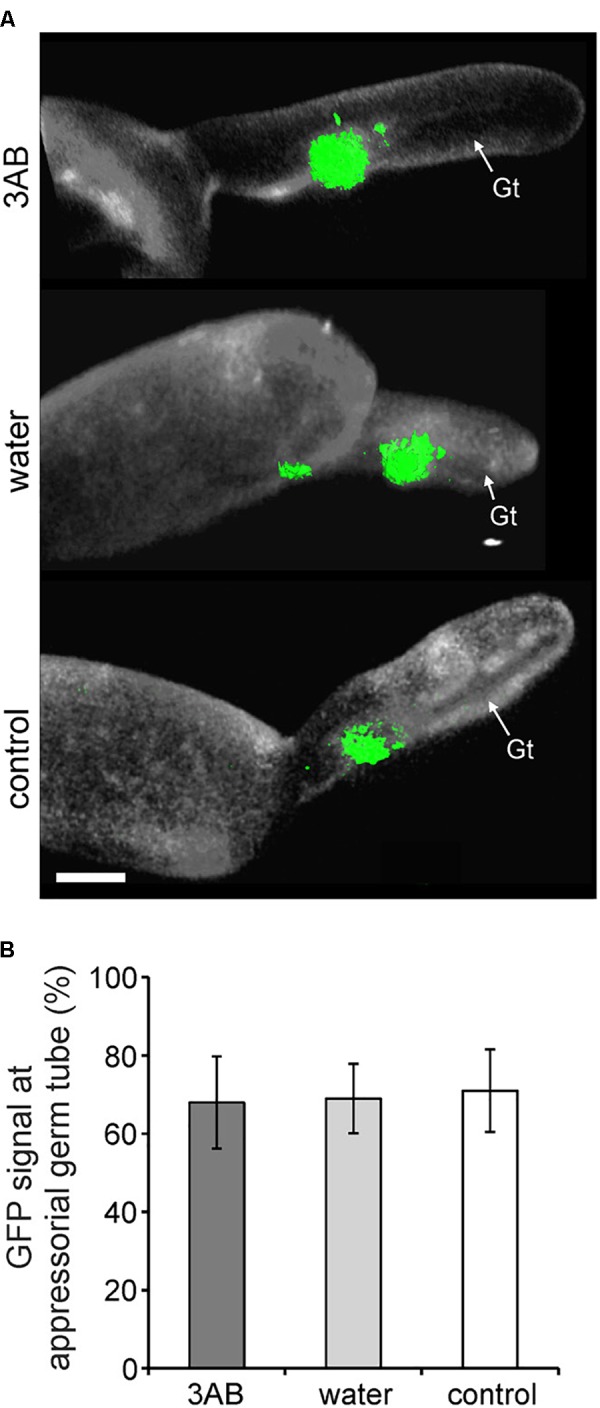
3AB does not detectably alter PMR4-GFP translocation to sites of attempted powdery mildew infection. Three-week-old overexpression line *35S:PMR4-GFP* was inoculated with a compatible powdery mildew (*G. cichoracearum, Gc*) isolate 2 h post- after infiltration with 10 mM 3AB. Leaves infiltrated with water served as mock control, leaves without infiltration as infection control. **(A)** Localization of the GFP-tagged callose synthase PMR4 at appressorial germ tubes (Gt) at 6 h post-inoculation (hpi). Representative micrographs are 3D projections taken by confocal laser-scanning microscopy. Green color assigned to GFP (green fluorescent protein)-emitted fluorescence, white color to aniline blue-stained (1,3)-*β*-glucan of the *Gc* cell wall. Scale bar = 5 μM. **(B)** Quantification of PMR4-GFP translocation to appressorial germ tubes at sites of attempted *Gc* penetration at 6 hpi. ANOVA significant differences were not detected between samples. Error bars represent ± SD; *n* = 25 of four independent leaves.

Utilizing the 35S:PMR-GFP overexpression line, we sought to identify if the relocalization of PMR4-GFP protein to sites of powdery mildew infection is altered by 3AB. We found no such evidence as the presence of GFP signal was observed similarly whether in the presence or absence of 3AB (Figure 7).

### 3AB Breaks the Complete Penetration Resistance of the PMR4 Overexpression Line

As previously reported, overexpression of PMR4 results in complete penetration resistance against powdery mildew (Ellinger et al., 2013). By 24 hpi, haustorium formation occurs and provides an indication of penetration success. As was also previously reported (Ellinger et al., 2013), the weak constitutive increase in resistance caused by loss of PMR4 (Nishimura et al., 2003) caused intermediate reduction in penetration success (Supplementary Figure S5). 3AB did not detectably impair that weak constitutive resistance of *pmr4* mutant plants. However, consistent with the role of callose in resistance to powdery mildew penetration (Ellinger et al., 2013), and the capacity of 3AB to block MAMP-induced callose inhibition, the complete penetration resistance of the PMR4 overexpression line was impaired by 3AB (Supplementary Figure S5). No evidence of penetration success was detected in PMR4 overexpression controls, while we observed instances of penetration success and haustorium formation in the 3AB-treated PMR4 overexpression leaves.

## Discussion

Poly(ADP-ribosyl)ation is best characterized for its roles in DNA damage repair, cell death, chromatin remodeling, and transcriptional regulation in mammals (Schreiber et al., 2006; Gibson and Kraus, 2012). Roles for poly(ADP-ribosyl)ation in plant immunity have also become increasingly well-documented (Adams-Phillips et al., 2008, 2010; Briggs and Bent, 2011; Feng et al., 2015, 2016a,b; Song et al., 2015; Briggs et al., 2017). Despite the roles of poly(ADP-ribosyl)ation in plant immunity demonstrated by other means such as mutational studies, the present work shows that the inhibition of MAMP-induced callose by the PARP inhibitor 3AB is independent of poly(ADP-ribosyl)ation. While 3AB and the structurally related 3MB block flg22- and elf18-induced callose deposition (Figure 3 and Adams-Phillips et al., 2008), we found that other more specific PARP inhibitors, such as INH_2_BP and PJ-34, do not. Moreover, *parp1parp2parp3* knockout mutants still produce flg22-induced callose at close to wild type levels. The activity of 3AB as a PARP inhibitor and an inhibitor of poly(ADP-ribosyl)ation has been widely and conclusively documented in animal and plant studies (present study and Chen et al., 1994; Panda et al., 2002; Virag and Szabo, 2002; Rouleau et al., 2010). However, despite common statements about 3AB as a specific inhibitor of poly(ADP-ribosyl)ation, several off-target impacts have been reported in mammalian systems. For instance, 3AB protects primary human keratinocytes from UV-induced cell death, but this phenotype was not reproduced with the inhibitor PJ-34 or with silencing of PARP1, indicating a poly(ADP-ribosyl)ation independent mechanism (Lakatos et al., 2013). In other studies 3AB has been shown to inhibit protein kinase C (Ricciarelli et al., 1998), certain cytochrome P450s (Eriksson et al., 1996), and to act as a hydroxyl radical scavenger (Czapski et al., 2004). Our study adds to the list of processes separate from poly(ADP-ribosyl)ation that are targeted by 3AB, demonstrating inhibition of MAMP-induced callose deposition as a new off-target effect of 3AB in plants. The present work also raises a question for future study: if not poly(ADP-ribosyl)ation, what pathways or proteins does 3AB act on to disrupt MAMP-induced callose synthesis?

Despite known non-specific impacts, 3AB is still frequently used as an inhibitor of poly(ADP-ribosyl)ation. Numerous Arabidopsis studies have utilized 3AB, where it helped implicate roles for poly(ADP-ribosyl)ation in the regulation of apoptosis, circadian rhythms, and stress responses (e.g., Tian et al., 2000; Panda et al., 2002; Adams-Phillips et al., 2008, 2010; Ishikawa et al., 2009; Schulz et al., 2012). Given this possibility of non-specific targets, reports utilizing 3AB to demonstrate a role for poly(ADP-ribosyl)ation in a given process should be treated with caution and should be accompanied by other inhibitors or, preferably, genetic evidence through the use of *parp* knockout lines (e.g., Song et al., 2015).

**FIGURE 8:**
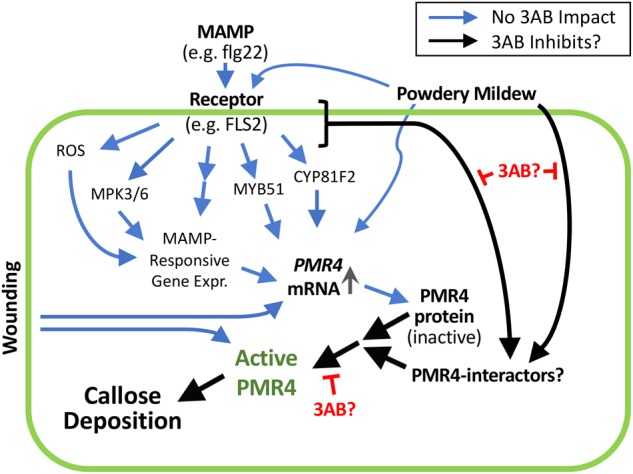
3AB reveals the presence of currently unknown flg22-inducible signaling mechanisms that activate callose synthesis. These signaling mechanisms are separate from the flg22-induced signals that elevate *PMR4* mRNA and protein levels, and separate from the wound-inducible signals that activate callose synthesis activity. Prospective scheme is based on present results and those of Clay et al. (2009) and Adams-Phillips et al. (2010). Postulated black arrow pathways are impacted by 3AB; the blue arrow pathways are not detectably impacted by 3AB. Black arrow from MAMP receptor may be absorbing signals downstream of processes shown to not be detectably impacted by 3AB, or from one or more independent pathways. In addition to blocking flg22-induced callose deposition, 3AB also reduces (but does not eliminate) the % of powdery mildew infection sites at which callose deposition is detectable. The existence of PMR4-interacting proteins is hypothesized; 3AB is postulated to inhibit components of the PMR4 complex other than PMR4 itself that are required for PMR4 complex formation and full enzymatic activity. Promising targets for future studies might include proteins that physically interact with PMR4 including any kinases or phosphatases that alter the phosphorylation status of PMR4, proteins that influence PMR4 subcellular localization, or other proteins that post-translationally modify PMR4.

We investigated the hypothesis that the lack of flg22-induced callose in the presence of 3AB could be explained by reduced PMR4 protein abundance. We instead observed that 3AB increases PMR4 protein abundance after flg22 treatment, despite a near-complete absence of new callose deposition. 3AB treatment along with flg22 does not cause increased abundance of PEN1 or PEN3, two proteins indirectly associated with callose deposition (Collins et al., 2003; Stein et al., 2006), and 3AB does not detectably alter flg22-induced *PMR4* mRNA abundance. The presence of more abundant PMR4 protein but no callose production in response to flg22 suggests that 3AB is impacting the localization or subsequent activation of the PMR4 protein (Figure 8). Importantly, the inhibitory activity of 3AB has specificity for MAMP-induced callose, because levels of wound-induced callose (which is also produced via PMR4) are not altered by 3AB. Moreover, the 3AB-induced increase in PMR4 abundance after flg22 treatment is not due to generic inhibition of callose synthase activity, because 3AB does not block wound-induced callose deposition and because an increase in PMR4 abundance was not observed when flg22-induced callose deposition was blocked by the callose synthase inhibitor 2-DDG. Together, the above findings with 3AB reveal the presence of flg22-inducible signaling mechanisms that activate callose synthesis activity separate from the flg22-induced signals that elevate PMR4 mRNA and protein levels, and separate from the wound-inducible signals that activate callose synthesis activity. One possible model summarizing these findings is presented in Figure 8. Blue arrows represent processes that are not blocked by 3AB while black arrows represent hypothesized processes whose inhibition is suggested by these 3AB studies. Additional aspects of the model are discussed below.

In previously published work, 3AB treatment did not alter flg22-induced *MYB51* or *CYP81F2* gene expression, suggesting that 3AB blockage of MAMP-induced callose is likely to be independent of and/or downstream of the *MYB51*/ethylene-dependent and *CYP81F2*/indole-3-glucosinolate pathways (Adams-Phillips et al., 2010). Addition of salicylic acid (SA) or benzothiadiazole (BTH; a chemical analog of SA) does not induce callose deposition in Arabidopsis seedlings, but addition of SA or BTH to flg22-treated seedlings rescued 3AB blockage of flg22-induced callose deposition, and did so independent of *NPR1* (Adams-Phillips et al., 2010). Callose deposition was still elicited by flg22 treatment in *nahG^+^* (salicylate-degrading) and *sid2^-^* (salicylate biosynthesis-defective) plants that have greatly reduced SA production (Adams-Phillips et al., 2010), as was also observed by Clay et al. (2009), which suggests that SA is not required for flg22-induced callose deposition. Taken together, these previous SA results could suggest that 3AB interferes with an SA-dependent, NPR1-independent callose pathway upstream of SA biosynthesis or, more likely, that in the presence of flg22, exogenous application of SA or BTH can activate an independent pathway and bypass the flg22-induced callose deposition pathway that is blocked by 3AB (Adams-Phillips et al., 2010).

We considered disruption of PMR4 localization at the plasma membrane or relocalization of PMR4 to the site of pathogen attack as possible targets of 3AB (Bohlenius et al., 2010; Nielsen et al., 2012; Ellinger et al., 2014). We constructed *PMR4pro:PMR4-GFP* lines that successfully rescued the *pmr4* mutant for flg22-induced callose deposition, as one possible avenue to investigate the MAMP- or pathogen-induced relocalization of the PMR4 in the presence and absence of 3AB. However, GFP fluorescence was not detectable above background in those lines using a sensitive Zeiss Elyra PS1 confocal microscopy system, possibly due to the low expression of *PMR4* from the *PMR4* promoter. As such, we utilized the 35S:PMR4-GFP overexpression line as an alternative approach. We also utilized a compatible isolate of powdery mildew (*G. cichoracearum*), a pathogen that forms localized infection pegs and has been used in multiple previous studies of pathogen-induced callose deposition (Ellinger and Voigt, 2014). In addition to blocking flg22-induced callose deposition, 3AB also reduced the % of *G. cichoracearum* infection sites at which callose deposition was detectable. While 3AB does not appear to inhibit transport of PMR4-GFP to sites of powdery mildew infection, we cannot firmly conclude that 3AB has no impact on PMR4-GFP relocalization, given that the impact of 3AB on powdery mildew-responsive callose is only partial as compared to MAMP treatment. Notably, a higher concentration of 3AB was required to impact powdery mildew-induced callose deposition (10 mM compared to 1 mM). This may result from powdery mildew more strongly activating callose responses than flg22, or from powdery mildew activating multiple signaling pathways that promote callose deposition, only some of which are blocked by 3AB.

3-Aminobenzamide could be inhibiting components of the PMR4 complex other than PMR4 itself that are required for PMR4 complex formation and full enzymatic activity. Promising targets for future studies might include proteins that physically interact with PMR4, kinases or phosphatases that alter the phosphorylation status of PMR4, or other proteins that post-translationally modify PMR4. Much remains to be understood about the regulation of PMR4 and callose deposition following pathogen perception, particularly as it contrasts with wound-induced callose deposition, and 3AB provides a useful tool for this research. Studies with 3AB have revealed the presence of one or more presently unknown pathways that regulate PMR4 activity and MAMP-induced or pathogen-induced callose deposition in plants.

## Data Availability

The raw data supporting the conclusion of this manuscript will be made available by the authors, without undue reservation, to any qualified researcher.

## Author Contributions

BK, JS, and AB conceived the original study and research plans. BK performed most of the experiments. JS, JN, and CV performed additional experiments. All authors designed the experiments and analyzed the data. BK and AB wrote the article with contributions from all of the authors.

## Conflict of Interest Statement

The authors declare that the research was conducted in the absence of any commercial or financial relationships that could be construed as a potential conflict of interest.
