# Supplementary material for: 3-Aminobenzamide Blocks MAMP-Induced Callose Deposition Independently of Its Poly(ADPribosyl)ation Inhibiting Activity
Source: Front Plant Sci. 2018 Dec 19;9:1907. doi: 10.3389/fpls.2018.01907 (PMC6305757; doi:10.3389/fpls.2018.01907)
Supplement: Supplementary file 1 [file Data_Sheet_1.PDF]

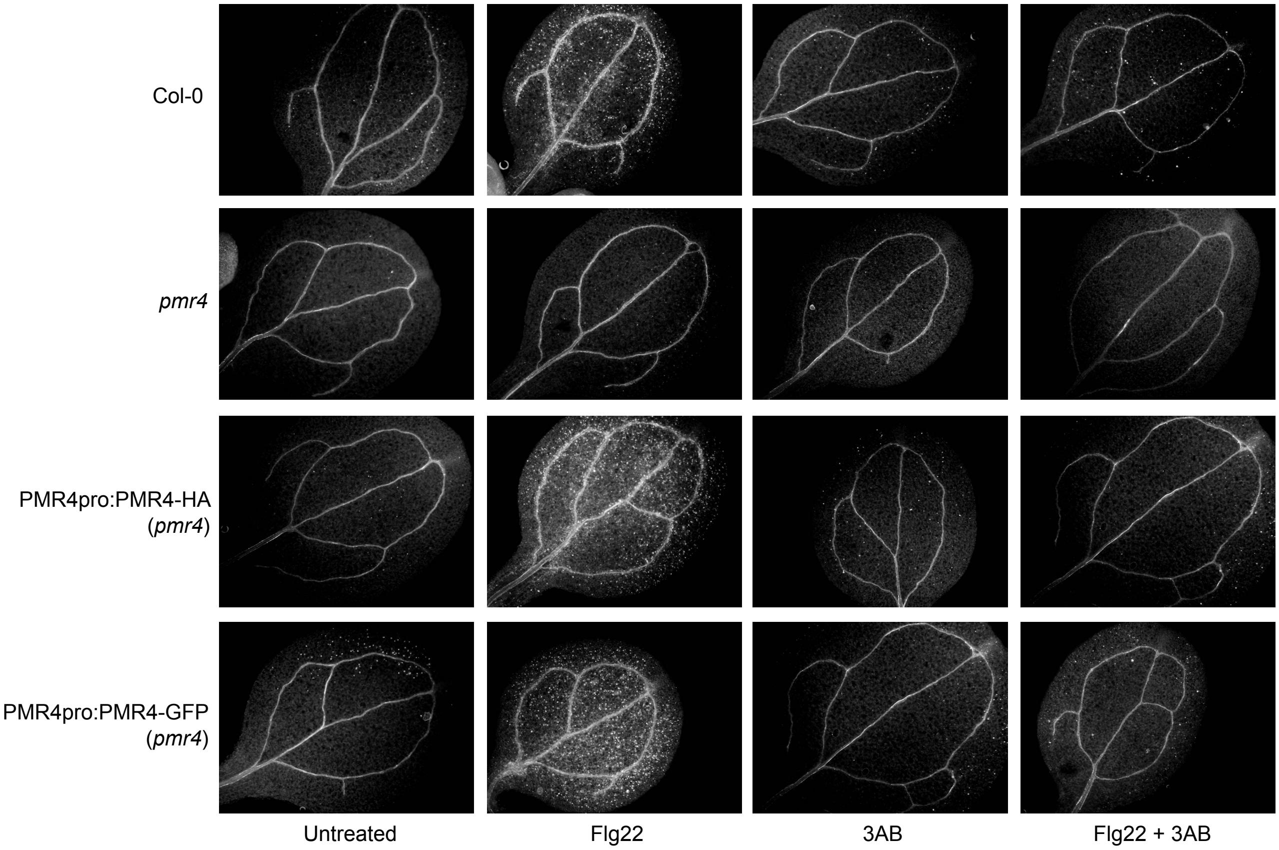

Supplemental Figure S1. *PMR4pro:PMR4-HA* and *PMR4pro:PMR4-GFP* lines successfully complement the *pmr4* knockout mutant. Representative images of Col-0 wild type, *pmr4* mutant, and PMR4-HA or PMR4-GFP lines (transformed in the *pmr4* mutant background) cotyledons untreated or treated with flg22, 3AB, or flg22+3AB.

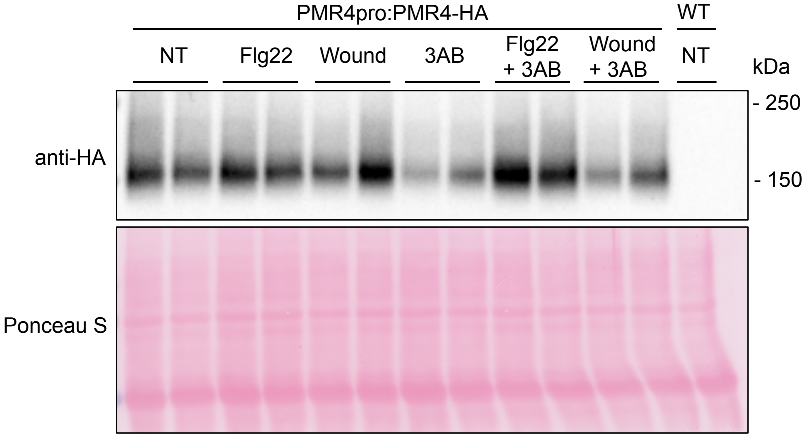

Supplemental Figure S2. Unlike flg22 treatment, wounding in the presence of 3AB does not increase PMR4 protein abundance. Total protein extracts from *PMR4pro:PMR4-HA* seedlings analyzed by immunoblotting with an anti-HA antibody. Two independent plant samples are shown for each treatment. Equivalent loading of lanes was verified using Ponceau S stain. NT: no treatment; WT: Col-0 wild-type negative control.

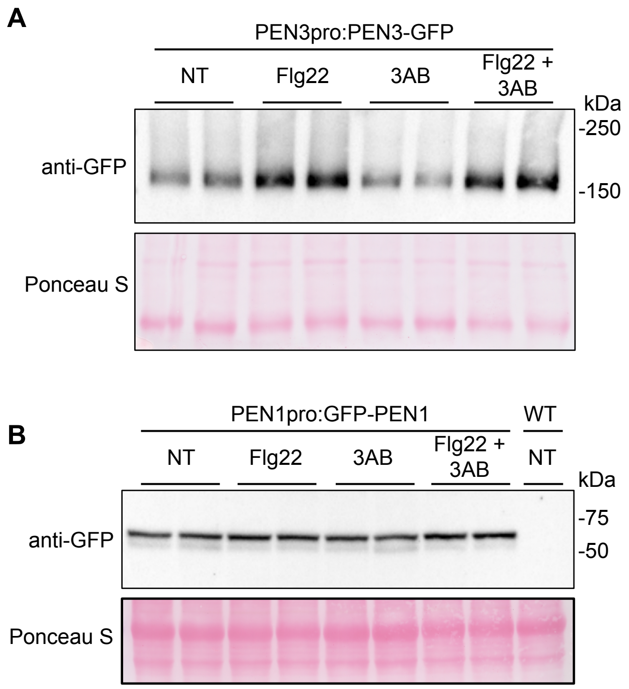

Supplemental Figure S3. Unlike PMR4-HA, 3AB does not alter PEN3-GFP or GFP-PEN1 protein abundance in response to flg22. Total protein extracts from (A) PEN3-GFP or (B) GFP-PEN1 seedlings were analyzed by immunoblotting with an anti-GFP antibody. Two independent plant samples are shown for each treatment. Equivalent loading of lanes was verified using Ponceau S stain. NT: no treatment: WT: Col-0 wild-type negative control.

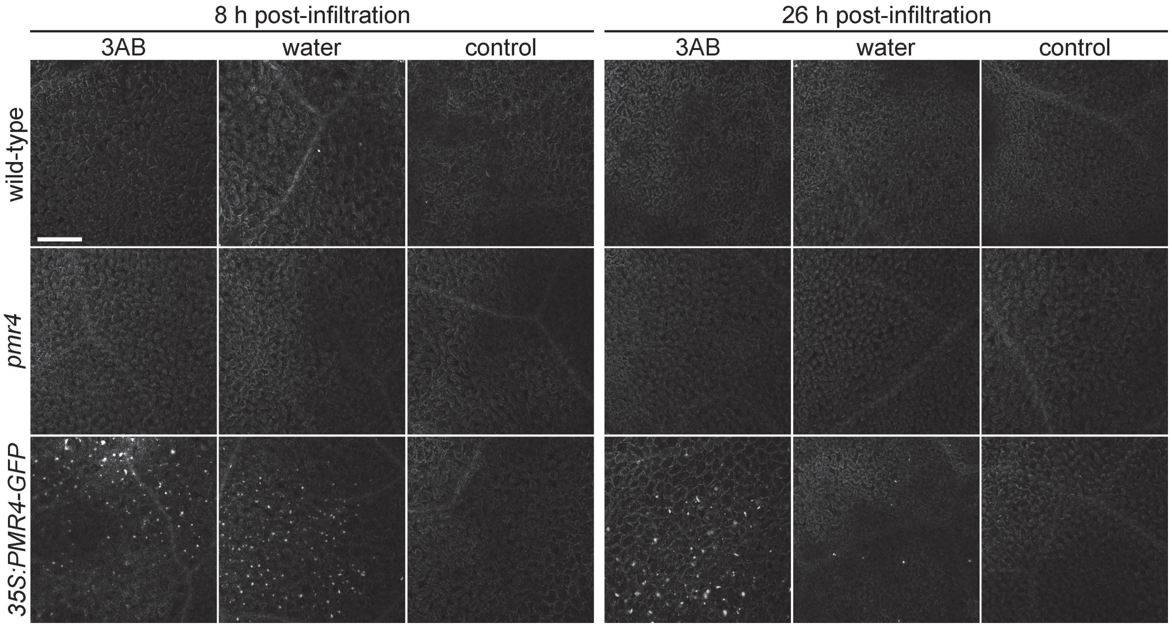

Supplemental Figure S4. Callose deposition is observed in leaves of *35S:PMR4-GFP* Arabidopsis after infiltration with water or 3AB. Three-week-old rosette leaves of the overexpression line *35S:PMR4-GFP*, *pmr4*, and wild-type were infiltrated with a 10 mM 3AB aqueous solution (3AB). Water was infiltrated to serve as a mock control, untreated leaves as negative control. Representative micrographs are shown, taken with a confocal laser scanning microscope to image leaves harvested 8 h and 26 h post-infiltration and stained with aniline blue. Fluorescence indicates callose deposition. Scale bar = 200 µm.

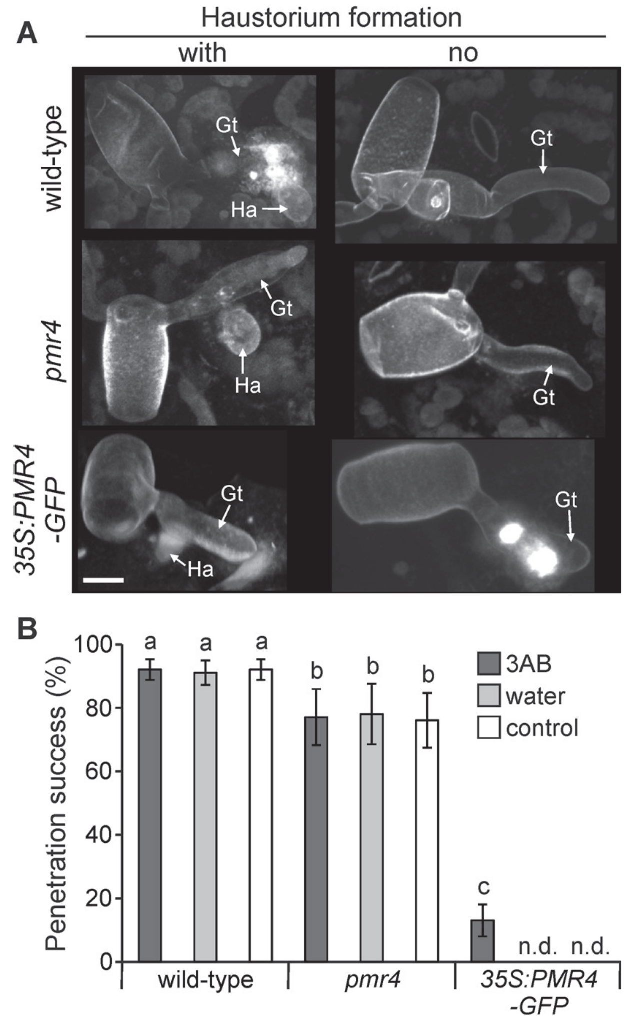

Supplemental Figure S5. 3AB treatment increases powdery mildew penetration success on *35S:PMR4-GFP* plants. Three-week-old overexpression line *35S:PMR4-GFP*, *pmr4* and wild-type plants were inoculated with a compatible powdery mildew (*G. cichoracearum*, Gc) isolate 2 h after infiltration with 10 mM 3AB. Leaves infiltrated with water served as mock control, leaves without infiltration as infection control. **(A)** Micrographs are representative of successful (with) and unsuccessful (no) Gc haustorium formation (Ha, fluorescence by aniline blue staining) at appressorial germ tubes (Gt) at 24 h post-inoculation (hpi). Micrographs are 3D projections taken by confocal laser-scanning microscopy. Scale bar = 10 µM. **(B)** Quantification of penetration success by haustorium formation at appressorial germ tubes as indicated by successful plant cell entry at 24 hpi. Treatments that do not share same letter are significantly different ($P < 0.05$ by Bonferroni-Holm's test). Error bars represent ± SD; n = 50 of 4 independent leaves; n.d.: haustorium formation not detected..
